# Functionalization of Peptides via Radical Arylation

**DOI:** 10.1002/cbic.70558

**Published:** 2026-10-09

**Authors:** Daniel R. Troeger, Markus R. Heinrich

**Affiliations:** ^1^ Department of Chemistry and Pharmacy Pharmaceutical Chemistry Friedrich‐Alexander‐Universität Erlangen‐Nürnberg Erlangen Germany

**Keywords:** arylation, cysteine, peptides, radical reactions, selenocysteine, tryptophan, tyrosine

## Abstract

Among the manifold methods for the functionalization of peptides known today, radical chemistry has so far only rarely been exploited to introduce structural modifications on this challenging type of substrate. In this short review, we give an overview showing that, especially, radical arylation reactions can represent attractive and powerful tools to access functionalized peptides under mild and biocompatible conditions where no protecting group strategy is required. Besides offering new opportunities to directly synthesize functionalized peptides for diverse biological applications, including drug development, it is also our intention to give deeper insight into aryl radical generation and aryl radical reactivity, which can, in a wider sense, be highly useful for future developments in the field of the selective radical functionalization of biomacromolecules.

## Graphical abstract

Radical chemistry has only rarely been applied to peptide functionalization. This short review highlights radical arylation as an attractive approach to access functionalized peptides under mild, biocompatible conditions without protecting groups. In addition, fundamental aspects of aryl radical generation and reactivity are discussed, providing guidance for the future development of selective radical functionalization of biomacromolecules.

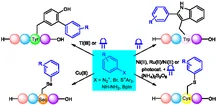

## 1 Introduction

Owing to their high reactivity toward many substrates, aryl radicals are commonly not considered reactive intermediates of choice when it comes to the selective functionalization of complex biomolecules. The reactivity of aryl radicals is, however, not a general feature but has to be assessed in relation to the individual functional groups occurring in the target molecule. While aryl radicals will, for example, lead to rapid hydrogen abstraction from a C─H unit in *α*‐position of an aliphatic ether, thus making tetrahydrofuran an unsuitable solvent for most radical arylations, hydrogen abstraction from the O─H bond of water is negligibly slow [1]. This is due to the background of relatively slow hydrogen atom transfer (HAT) from many polar, electron‐poor X─H bonds to electrophilic aryl radicals, where the most important heteroatoms X are oxygen and nitrogen. As a direct result, water represents a nearly ideal solvent for radical arylations due to its low reactivity. Moreover, and based on the same effect, most aliphatic alcohols, amines, ammonium salts, and amides, as well as carboxylic acids and carboxylates—as they are often present in biomolecules—display comparably low reactivity toward aryl radicals. Taken together, these considerations suggest that aryl radicals—despite their commonly assumed reactivity—may be better suited for the functionalization of biomolecules.

Focusing on the modification of peptides, which is the main topic of this short review, a key aspect is the stability of the peptide backbone toward aryl radicals. Such stability is not only required to direct radical reactions to the side chains of the amino acids but also to prevent undesired side reactions such as epimerization or substitution at one or the other stereocenter. While hydrogen abstraction from the amide subunits can be expected to be slow due to the above‐mentioned polarity mismatch between the polar and electron‐poor N─H bond and the electrophilic aryl radical, the less polar C─H bond in *α*‐position as well as C─H bonds in *β*‐position may remain potentially vulnerable at first sight. In this context, it is useful to refer to a seminal work by Easton [2], in which the backbone as well as the adjacent C─H bonds in the side chains of amino acids and peptides displayed peculiar stability toward hydrogen atom transfer. These effects were in particular observed for strongly electrophilic radicals such as chlorine and the hydroxyl radical. Although common aryl radicals are not as strongly electrophilic as chlorine or hydroxyl radicals [3], they are considered electrophilic when compared to other carbon‐centered radicals [4], which can serve as an explanation for the reasonable overall stability of the peptide backbone and proximate C─H bonds. In this context, it is important to note that late‐stage C─H functionalization at amino acid side chains can nevertheless be achieved if suitably electrophilic radicals other than the aryl radical are chosen. This has been demonstrated for several examples where the initial HAT from the respective C─H bond plays a key role [5, 6, 7, 8]. Against a more general background, the overall stability of the peptide backbone toward electrophilic radicals represents a highly important feature since it provides a rationale for the ability of amino acids and peptides to remain largely stable in nature, although being permanently exposed to radicals. In addition, it provides an explanation for enzymes being able to catalyze radical reactions without suffering from constant degradation.

Taking into account these general considerations, peptides no longer appear to be such unfavorable substrates for radical functionalization, in particular, if the structural modification is intended to occur in one of the side chains. Through the examples summarized in this short review, we will show which side chains are especially suitable for radical arylations and to what extent selectivity can be achieved over other amino acids and their respective side chains. From a chemical perspective, radical arylations of complex biomolecules [9] represent a valuable extension of the common synthetic chemist’s toolbox, which often largely relies on a number of well‐established ionic and transition‐metal‐catalyzed reactions.

Before presenting illustrative examples for radical arylation of peptides, it is necessary to define the scope of this short review. This review focuses on the direct radical arylation of naturally occurring proteinogenic amino acid residues within peptides and proteins. Several alternative and synthetically related approaches, which have been summarized in recent review articles [9, 10, 11, 12], are therefore not considered in detail. In particular, radical arylations of non‐proteinogenic amino acids and amino acid‐derived functionalities are beyond the scope of the present review. This distinction is particularly relevant for dehydroalanine, whose electron‐deficient C═C double bond can serve as a highly reactive site for radical arylation [13, 14]. Although such methods constitute an important strategy for peptide modification, they represent a different approach due to the targeting of a non‐proteinogenic amino acid.

The extensive field of organometallic arylation methods is likewise not covered comprehensively, although these transformations provide an important point of comparison for the radical approaches discussed below. Numerous transition‐metal‐catalyzed and metal‐mediated reactions have been developed for the arylation of individual amino acid residues, peptides, and peptide‐derived substrates. Examples include the arylation of cysteine (Cys) [15, 16, 17, 18, 19], lysine (Lys) [20], histidine (His) [21, 22], alanine/isoleucine/valine (Ala/Ile/Val) [23, 24], proline (Pro) [25], tryptophan (Trp) [26, 27], and tyrosine (Tyr) [28, 29, 30], as well as methods enabling arylation at the N‐terminus of peptides [31]. These approaches demonstrate that a broad range of amino acid side chains and peptide positions can be accessed by organometallic or related coupling strategies. They therefore provide powerful and often highly selective alternatives to radical arylation, but their comprehensive discussion would go far beyond the scope of this short review. Depending on the particular reaction design, organometallic and metal‐mediated arylation methods can benefit from the well‐established and predictable reactivity of organometallic intermediates and from the broad range of available aryl electrophiles and coupling partners. In particular, catalyst and ligand design can provide considerable control over chemo‐ and regioselectivity. On the other hand, such methods may require pre‐functionalized reaction partners, specifically engineered peptide substrates, protecting‐group strategies, or reaction conditions that are not compatible with all functionalities present in complex peptides. Coordination of peptide residues to the metal catalyst and competing metal‐mediated reactions can further complicate the direct functionalization of unprotected peptides.

Radical arylation of peptides, as discussed below, offers a complementary set of opportunities. Aryl radicals possess a distinct reactivity profile that can allow direct functionalization of otherwise challenging substrates without requiring the pre‐installation of a conventional organometallic coupling handle. Moreover, the relatively slow HAT from many polar X─H bonds to electrophilic aryl radicals can provide surprisingly high compatibility with the functional groups commonly encountered in peptides. This feature enables radical arylations under aqueous conditions and, in some cases, without protecting groups, transition‐metal catalysts, or additional reagents. The possibility of generating aryl radicals from readily accessible precursors and of exploiting photochemical initiation further expands the range of reaction conditions available for peptide modification. These advantages are, however, accompanied by characteristic limitations. In contrast to many well‐established organometallic coupling reactions, radical reactions are governed by a complex network of competing HAT, electron transfer, radical addition, recombination, and radical trapping processes. Consequently, the chemoselectivity of an aryl radical cannot necessarily be predicted solely from the presence of a potentially reactive functional group and may depend strongly on the complete molecular environment and reaction conditions. Radical arylation should therefore not be regarded as a replacement for classical organometallic peptide functionalization, but rather as a complementary strategy that can exploit a different reaction manifold and thereby provide access to transformations that are difficult to achieve by conventional ionic or organometallic chemistry.

A further distinction concerns radical methods for peptide cyclization. A variety of radical and radical‐type reactions have also been explored for peptide cyclization [32, 33, 34, 35, 36], but these approaches involve radical species other than aryl radicals. To the best of our knowledge, aryl radical‐mediated peptide cyclization has not yet emerged as an established general strategy. Such transformations are therefore not considered a separate topic in this review, which instead focuses on the aryl radical‐mediated installation of aryl groups onto amino acid residues.

Finally, it needs to be mentioned that additional methods for radical arylation exist, which have so far not been applied to peptides but only to single amino acids. Typically, and as is the case for most of the transformations discussed below, the related radical arylations have been developed and optimized using the respective single amino acids, and if suitable reaction conditions could be found, these methods were later applied to peptidic substrates. An illustrative example is the radical arylation of the imidazole ring of histidine, which is comparably slow—as it will also be shown within the discussion of the methods outlined below. For this reason, the arylation of the isolated amino acid is known for histidine [37], but an extension to peptidic substrates would likely be complicated by numerous competing side reactions. Another highly attractive transformation, namely, the decarboxylative arylation of *α*‐amino acids via photoredox catalysis by McMillan [38], represents a borderline case in the sense that radical anion‐type aryl species occur as intermediates instead of classical aryl radicals. Although only functionalizations of single amino acids have been described in this work, they could potentially be useful for the modification of the C‐termini of peptides.

## 2 Radical Functionalization of Peptides

Numerous methods have been reported for the generation of aryl radicals using a variety of different precursors acting as radical sources [39]. Among these, diazonium salts and related reagents—despite their early discovery—still play a major role [40]. In this short review, we will first discuss two functionalizations relying on the stoichiometric addition of a reductant or an oxidant to trigger radical generation. In the second section, six radical arylations are summarized, which are driven by visible light irradiation, either in the presence or in the absence of one or more catalysts.

### 2.1 Conventional Methods

The generation of aryl radicals from aryldiazonium salts by using the reductant titanium(III) chloride has the general advantage that the related reactions can be carried out in diluted aqueous hydrochloric acid, which is, as discussed above, a well‐suited solvent for radical arylations due to the very limited HAT. After preliminary studies on the arylation of tyrosine [41, 42], where essentially no protecting groups are required due to the low reactivity of carboxylic acids, protonated aliphatic amines, and phenolic hydroxy groups toward aryl radicals under acidic aqueous conditions, the method was then transferred to the arylation of peptides (Scheme 1) [43]. Regarding the phenolic hydroxy group, it is important to note that phenols are typically very effective antioxidants and radical scavengers when used in non‐aqueous solvents of low polarity, comparable to the natural antioxidant tocopherol for lipophilic environments, but the ability to stabilize radicals via HAT from a phenol is strongly decreased in water or water‐containing solvent mixtures [44].

**SCHEME 1:**
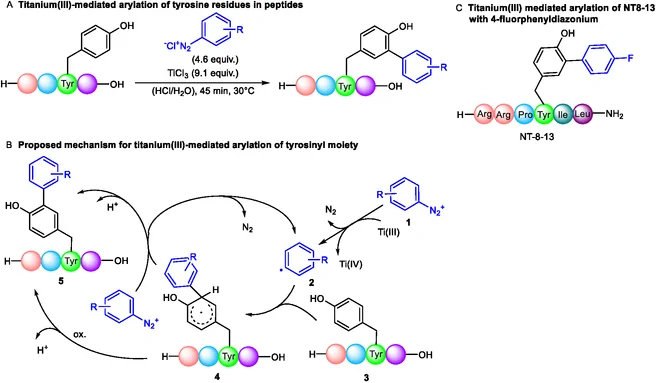
Titanium(III)‐mediated reductive arylation of peptides [43].

Taking a closer look at the underlying reaction mechanism, the radical arylation starts with the titanium‐mediated, reductive generation of aryl radical **2** from diazonium ion **1**. Arylation of the peptidic substrate **3** at the 3‐position of phenol leads to a cyclohexadienyl intermediate **4**. Oxidative rearomatization of **4** may readily occur through oxygen [45], but also in the sense of a chain mechanism whereby a new aryl radical **2** is generated from diazonium ion **1** along with the arylated peptide **5**. Competition experiments, which were carried out in addition to the main study, revealed that the radical arylation of the tyrosine side chain is faster than that of the aromatic amino acids histidine and phenylalanine but slower than the arylation of tryptophan. On this basis, it is not surprising that radical arylations of tryptophan can also be carried out efficiently, as will be described later in this short overview. Overall, the titanium‐mediated arylation shown in Scheme 1 enables straightforward access to peptides arylated at their tyrosine unit. A major drawback, however, was the large excess of titanium(III) chloride required for arylation, which complicates the work‐up and purification.

In contrast to the strongly acidic conditions described for the titanium(III)‐mediated arylations, the generation of aryl radicals can also be achieved under milder oxidative conditions starting from arylhydrazines. Using copper(II) as a stoichiometric additive to facilitate oxidation of the arylhydrazine in the presence of oxygen, effective arylations of selenocysteine‐containing peptides could be realized in an aqueous phosphate buffer at a pH value of 6 (Scheme 2) [46]. While unmodified selenocysteine (Sec) and cysteine (Cys) would not represent ideal targets for radical arylation due to competing HAT from the Se─H or S─H bond [47, 48], the corresponding diselenides (Se─Se bond) or mixed selenocysteine‐cysteine adducts containing a Se─S bond do not suffer from this undesired side reaction. As shown in Scheme 2B, the attack of the aryl radical **2**—previously generated from aryl hydrazine **6**—onto the Se─Se or Se─S bond of substrate **7** leads to the short‐lived adduct **8** undergoing Se─Se or Se─S bond cleavage to furnish the arylated peptide **9** along with a selenyl‐ or thiyl radical‐containing peptide **10** or **11**. Oxidative regeneration of substrate **7** from **10** and complexation of copper(II) ions close the reaction cycle.

**SCHEME 2:**
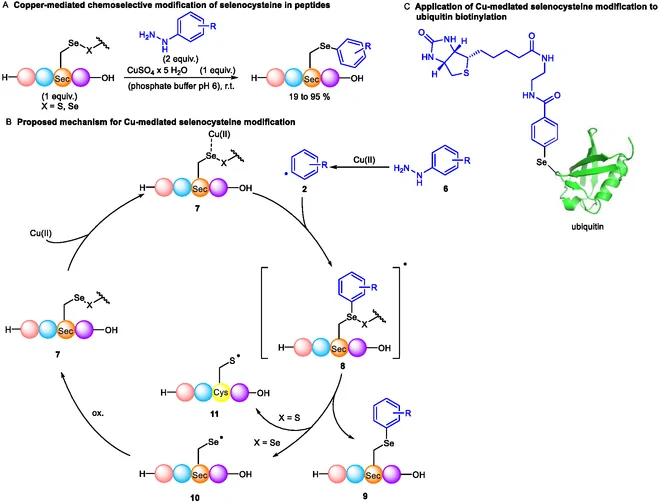
Copper‐mediated arylation of selenocysteine using arylhydrazines as radical precursors [46].

Notably, aryl radical attack in the key step may occur directly on the Se─Se or Se─S bond or on the copper ion bound to the selenium atom, whereby the formation of **9** would then proceed via reductive elimination of a copper ion or copper‐containing complex.

The main reason for these highly successful radical arylations, which is closely linked to the fact that Se─Se and Se─S bonds as well as the corresponding copper adducts represent very efficient traps for aryl radicals, can also be deduced from the observation that the aryl hydrazine **6** can be added to the reaction mixture in one batch. If the aryl radical attack onto the Se─Se or Se─S bond or on a complexed copper ion was not that fast, newly generated aryl radicals could as well abstract hydrogen atoms from remaining arylhydrazine, which would lead to an undesired formation of substituted benzenes. Moreover, the outstanding capabilities of Se─Se and Se─S bonds to act as radical traps are nicely underlined by the successful arylation of complex substrates such as ubiquitin (Scheme 2C).

A related reaction has been successfully applied to disulfide bonds in peptides (Scheme 3). In this case, aryl radicals generated from aryl pinacol boronates under mild aqueous conditions can react with disulfide bridges in peptides, yielding *S*‐arylated products [49]. Similar to Se─Se and Se─S bond arylations presented above, the absence of an X─H bond at the reactive sulfur center circumvents the competing HAT that often complicates the arylation of Cys and Sec residues in peptides. In particular, the reaction does not require photochemical initiation or a metal catalyst but proceeds in the presence of ammonium persulfate as a catalytic oxidant. Radical trapping and spectroscopic experiments point to the involvement of aryl radicals, although the detailed mechanism of this disulfide functionalization remains to be fully established. First applications of this method to proteins led to promising results regarding the selective functionalization of complex substrates.

**SCHEME 3:**
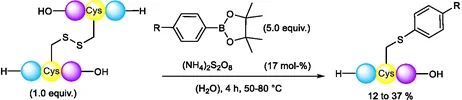
Metal catalyst‐free, ammonium peroxodisulfate‐mediated *S*‐arylation of disulfides using aryl pinacol boronates as an aryl radical precursor [49].

### 2.2 Photoinitiated Methods

The direct radical substitution on the sulfur atom of a cysteine side chain is typically complicated by competing HAT to the aryl radical [1]. As shown above for the even more reactive selenocysteine (Scheme 2), one option to circumvent this difficulty is to perform the radical arylation on the corresponding diselenide, which is still a reactive aryl radical scavenger but bears no hydrogen atom for abstraction. Another possibility to avoid undesired HAT is to primarily convert cysteine into a photosensitive diazosulfide. This strategy has been successfully used to achieve ^18^F‐fluoroarylation of glutathione (Scheme 4) [50].

**SCHEME 4:**
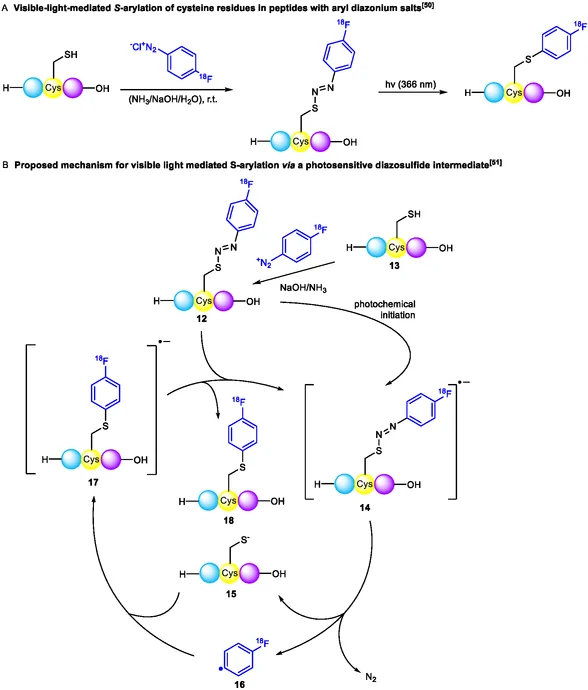
Visible‐light mediated *S*‐arylation of cysteine via a photosensitive diazosulfide intermediate [50, 51].

In the proposed mechanism (Scheme 4B), which is based on earlier studies on radical reactions of arylazosulfides [51], the glutathione‐derived azosulfide **12** was first obtained from glutathione (**13**) under strongly basic conditions. Photochemical initiation then converts azosulfide **12** into the short‐lived radical anion **14**, which cleaves into the thiolate **15**, nitrogen, and the 4‐[^18^F]fluorophenyl radical (**16**). Besides the pathway via aryl radical attack on thiolate **15** and the occurrence of a second radical anion **17**, formation of the 18F‐fluoroarylated glutathione **18** can also be rationalized via an attack of aryl radical **16** on azosulfide **12**, followed by rapid fragmentation and the generation of a new 4‐[^18^F]fluorophenyl radical (**16**) to propagate the radical chain (not shown).

Somewhat surprisingly, this otherwise attractive cysteine arylation strategy has not yet been directly employed beyond radiochemical labeling. Although competing azo coupling can be expected for tyrosine units, which are present as phenolates under the strongly basic conditions used for azosulfide formation, the arylation method has advantages due to its metal‐ and catalyst‐free character as well as its ability to avoid competing hydrogen abstraction.

More recently, the radical arylation of cysteine residues has been achieved in combination with the in situ diazotization of anilines and in the presence of the photocatalyst eosin Y under visible‐light irradiation (Scheme 5) [52]. While moderate to good yields could already be obtained for variously functionalized anilines in batch‐wise reactions, the transfer of the method to a flow reactor even improved the yields at residence times ranging from 30 to 150 s. Besides successful examples on the gram scale, the photocatalyzed cysteine arylation was further able to selectively functionalize a model peptide bearing further nucleophilic side chains.

**SCHEME 5:**
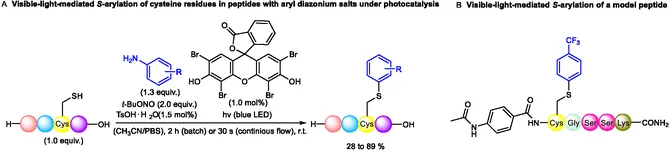
Photocatalyzed cysteine arylation combined with in situ diazotization of anilines [52].

Regarding a possible reaction mechanism, in situ diazotization of anilines with *tert*‐butylnitrite in the presence of catalytic amounts of acid can be expected to lead to *tert*‐butyl aryldiazo ethers and not primarily to free diazonium ions. Assuming nucleophilic substitution on the arylazo unit by the cysteine thiol, whereby *tert*‐butanol would be liberated, the actual arylation could occur starting from arylazosulfides as key intermediates, similar to the mechanism depicted in Scheme 4. In contrast to direct radical initiation from arylazosulfides, which requires UV light at a wavelength of 366 nm (c.f. Scheme 4), excitation of eosin Y with visible light also generates a reducing species capable of triggering the reductive cleavage of the azosulfide.

Compared to the generation of aryl radicals from diazonium ions, the use of aryl bromides as aryl radical precursors typically requires much stronger reducing conditions. As shown in Scheme 6, this general drawback of aryl bromides can be nicely overcome by using a combination of a nickel‐ and a ruthenium‐based photoredox catalyst (Scheme 6) [53]. Starting with photochemical excitation of the ruthenium catalyst, electron transfer from the bis(catechol) *tert*‐butylsilicate **19** liberates a *tert*‐butyl radical **20**, which transforms the cysteine‐containing peptidic substrate **21** into the corresponding thiyl radical **22** through HAT. The thiyl radical **22** is then readily trapped by a previously formed nickel(0) complex **23** to give the low‐valent nickel(I)‐cysteine complex **24**. Complex **24** is well‐suited for the oxidative addition of the aryl bromide **25**, thus leading to the key intermediate **26**, from which the arylated peptide **27** arises via reductive elimination of a nickel(I) bromide complex **28**.

**SCHEME 6:**
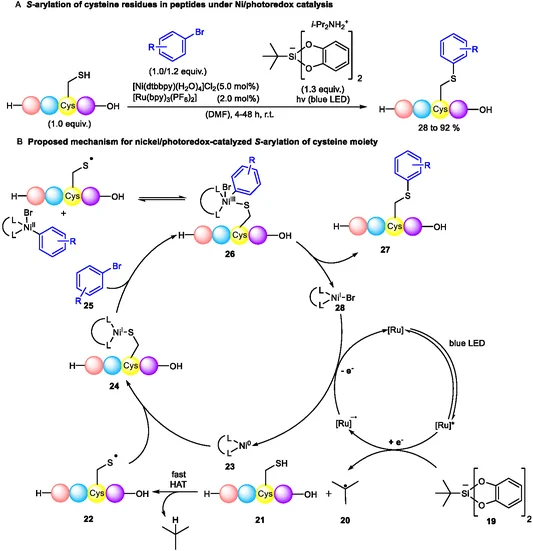
*S*‐arylation of cysteine‐containing peptides under nickel/photoredox catalysis [53].

As is evident from the reaction mechanism, this dual‐catalyzed arylation represents a certain borderline case since no free aryl radical is involved but only a thiyl radical **22** and an aryl–nickel complex with a reduced aryl radical character. As such borderline cases are, however, not uncommon, where the radical species is only more or less free and to a certain extent tamed through an equilibrium with, or complexation by, a corresponding metal atom, we nevertheless decided to include this arylation variant in the short overview. In addition to this method, and underlining the importance of these radical borderline cases, especially for cysteine arylation, a number of further metal‐catalyzed variants have been reported, whereby palladium [16, 17] and gold [18, 19] were used as catalysts.

Besides originating from aryldiazonium salts, aryl radicals may also be reductively generated from arylthianthrenium salts [54, 55]. This convenient access to aryl radicals has recently also been employed for cysteine *S*‐arylation, where substoichiometric nickel(II) ions were added to achieve the in situ formation of a peptide‐ and substrate‐derived photocatalyst **29** (Scheme 7) [56]. Upon reduction of the excited photocatalyst **30** by thianthrene **31**, a strongly reducing radical anion **32** is formed along with the thianthrenium cation **33**, whereby **32** triggers aryl radical generation from the aryl‐thianthrenium ion **34**. Addition of the aryl radical **2** to the peptidic thiolate anion **35** leads to the short‐lived, strongly reducing radical anion **36**, which is able to propagate the radical chain through a final single electron‐transfer step. Accompanied by the formation of the arylated peptide **37**, a new aryl radical **2** is liberated from the aryl‐thianthrenium ion **34**. Regarding the overall reaction conditions, the presence of diisopropylethyl amine not only ensures effective formation of the photoredox‐active nickel complexes **29**, but it further leads to a high degree of deprotonation of the cysteine S─H bond of the peptidic substrate **38** to give thiolate **35**. In turn, hydrogen abstraction from the cysteine S─H bond by the aryl radical, which represents a major undesired side reaction, can largely be suppressed. In addition to the aryl radical attack on the thiolate anion **35** to give **36**, which is proposed as the major pathway, alternative arylation on a nickel‐complexed cysteine residue cannot be fully excluded. In this case, and due to a presumably weaker complexation of the *S*‐arylated peptide **37** compared to the original substrate **35**, a subsequent ligand exchange on the nickel ion appears then to be likely.

**SCHEME 7:**
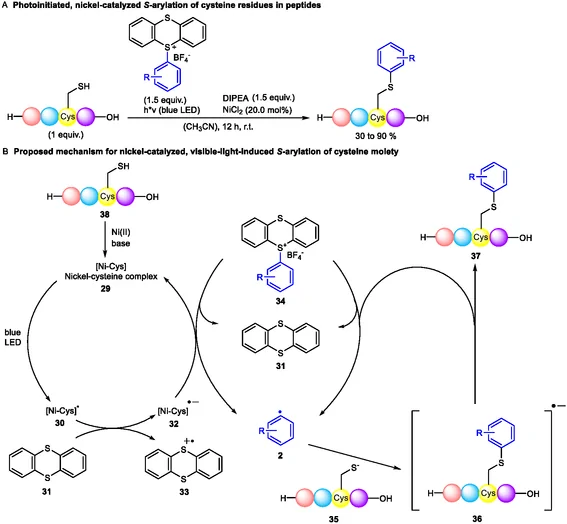
Photoinduced *S*‐arylation of cysteine‐containing peptides using an aryl‐thianthrenium salt as radical precursor [56].

In contrast to the in situ formation of the photoredox‐active nickel complex described above, the exploitation of charge–transfer (CT) complexes between aryldiazonium ions and electron‐rich subunits of the peptidic substrate does not require a particular additive to the reaction mixture but can also enable radical initiation (Scheme 8) [57]. While pioneering studies by Kochi [58] suggested that photoinduced aryl radical generation from aryl–aryl CT complexes is largely limited to electron‐poor aromatic diazonium ions, later investigations revealed that such additive‐free initiation can also be effective for combinations of less electron‐deficient aryldiazonium compounds with donor‐substituted arenes such as phenols or phenyl ethers [59]. This experimental background serves as a general rationale for the two additive‐free, photo‐induced arylations discussed in the final section of this overview, in which the two amino acids bearing electron‐rich aromatic side chains—tryptophan and tyrosine—were not only used for initiation via visible‐light irradiation of the corresponding diazonium‐based CT complexes but also in which the respective aromatic side chains served as target structures for the subsequent attack of the aryl radical.

**SCHEME 8:**
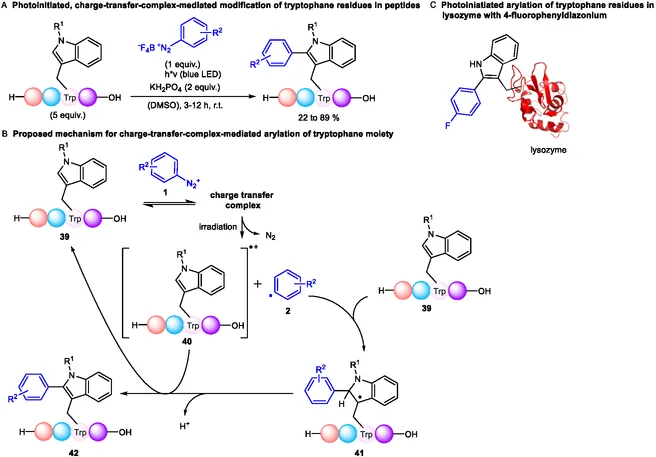
Photo‐induced, CT complex mediated arylation of tryptophan‐containing peptides using an aryldiazonium salt as radical precursor [57].

As shown in Scheme 8, the combination of aromatic diazonium salt **1** with a tryptophan‐containing peptide **39** will, in equilibrium, lead to the formation of photosensitive CT complexes. Irradiation of the reaction mixture by visible light will then trigger single‐electron transfer within the complex that results in a peptide‐derived radical cation **40**, nitrogen, and the required aryl radical **2**. Aryl radical attack on the tryptophan side chain of the peptidic substrate **39** at first provides an adduct radical **41**, which furnishes the arylated peptide **42** upon oxidative rearomatization. Single‐electron transfer in the rearomatization step may either occur to the previously formed radical cation **40** or to an aryldiazonium ion **1**, whereby the latter case leads to radical chain propagation as a new aryl radical is formed.

As already mentioned for the titanium‐mediated arylation of peptides (c.f. Scheme 1), the indolyl side chain of tryptophan represents the aromatic subunit in natural amino acids that is most reactive toward radical arylation. While this feature makes tryptophan an outstanding target for the attack of aryl radicals, when, for example, compared to tyrosine, phenylalanine, or histidine, the use of aryldiazonium salts in combination with tryptophan may be complicated to some extent by competing azo coupling of the diazonium ion to the electron‐rich indole core [60]. Nevertheless, the successful photoinduced and CT‐based radical arylation of the complex substrate lysozyme could be realized under the optimized conditions described in this study (Scheme 8C).

The formation of transient but sufficiently photosensitive CT complexes to enable radical initiation under visible light irradiation is also a key feature in the additive‐free arylation of tyrosine (Scheme 9) [61].

**SCHEME 9:**
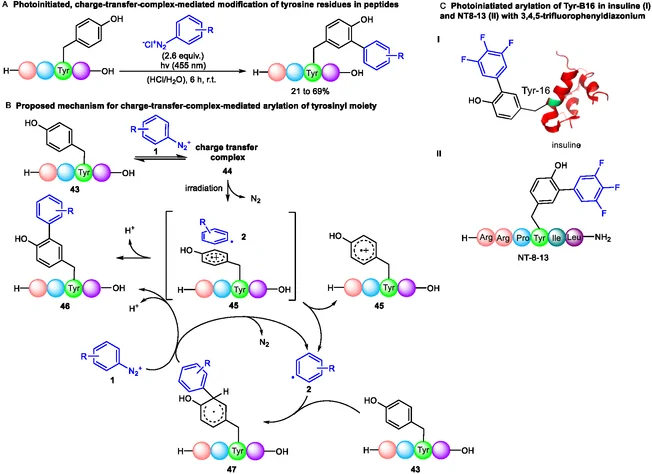
Additive‐free, photoinduced arylation of tyrosine‐containing peptides using aryldiazonium salts [61].

Similar to the reaction mechanism discussed above (c.f. Scheme 8), the peptidic, tyrosine‐containing substrate **43** and the aryldiazonium ion **1** are able to form visible‐light‐absorbing CT complexes **44** (Scheme 9B). Upon excitation, single‐electron transfer proceeds, and nitrogen is liberated along with the tyrosine‐derived radical cation **45** and aryl radical **2**. Based on control experiments on the reaction mechanism, direct radical recombination between **45** and **2** may occur to give the arylated peptide **46**, but the more likely pathway involves an intermolecular attack of the aryl radical on substrate **43**. Oxidative rearomatization of the cyclohexadienyl adduct **47** may proceed via electron transfer to **45**, by oxygen‐mediated oxidation [45], or via reduction of an aryldiazonium ion **1** to close the radical chain. While radical arylations of tyrosine subunits with aryldiazonium salts do not suffer from competing azo coupling when conducted under acidic conditions, a certain drawback is the higher reactivity of the tryptophan side chain toward aryl radicals.

Among the aryldiazonium salts investigated in the study on tyrosine arylation, the 3,4,5‐trifluorophenyldiazonium ion exhibited particular properties since an uncommon CT complex with 2:1 stoichiometry between the diazonium ion and tyrosine was observed. Besides facilitated photochemical initiation from these particular complexes—compared to the 1:1 CT complexes of typical aromatic diazonium salts—the 3,4,5‐trifluoro substitution pattern on the aromatic core enables further functionalization via nucleophilic aromatic substitution after performing tyrosine arylation. Synthetic usefulness was demonstrated by the successful arylations of Leu‐enkephalin amide as well as the complex substrate insulin (Scheme 9C).

## 3 Summary and Outlook

In summary, this overview of radical arylations of peptidic substrates demonstrates that mainly four amino acids are suitable targets for radical substitution (Table 1).

**TABLE 1:** Overview of reported peptide functionalizations via radical arylation and their corresponding reaction conditions.

Amino acid residue	Substrate(s)	Aryl radical precursor	Stoichiometric reagent, catalyst, photocatalyst or initiator	Solvent	Selectivity, limitations or special features	References
**Tyr**	Amino acids, peptides	Aryldiazonium salt	TiCl_3_	Aqueous HCl	Competition with Trp	[43]
	Amino acids, peptides,proteins	Aryldiazonium salt	Visible light	Aqueous HCl	Competition with Trp	[61]
**Cys**	Amino acids, glutathione	Arylazosulfide	UV light	Aqueous NH_3_, NaOH	Potential azo coupling	[50]
	Amino acids, peptides	Aniline (in situ diazotization)	[eosin Y] visible light	CH_3_CN/PBS^a^	Improved yield under continuous‐flow conditions	[52]
	Peptides	Arylbromide	[Ni]/[Ru] visible light	Dimethyl formamide	Radical/metal‐mediated pathway	[53]
	Peptides	Arylthianthrenium salt	[Ni]^b^ visible light	CH_3_CN	Requires thiolate for reactivity	[56]
	Peptides	Aryl pinacol boronates	(NH_4_)_2_S_2_O_8_	H_2_O	Requires S─S bond	[49]
**Sec**	Peptides, proteins	Arylhydrazine	CuSO_4_ O_2_	PBS^a^	Requires Se─Se/Se─S bond	[46]
**Trp**	Amino acids, peptides, proteins	Aryldiazonium salt	Visible light	Dimethyl sulfoxide	Potential azo coupling	[57]

a Phosphate‐buffered saline.

b Nickel(II)‐Cys complex in situ formation.

While all methods benefit from the peculiar stability of the peptide backbone toward the attack of electrophilic radicals, the side chains of cysteine, selenocysteine, tryptophan, and tyrosine represent suitably reactive sites for radical arylation. In the case of cysteine and selenocysteine, the actual site of substitution has to be present in a way that prevents competing HAT from the S─H or Se─H bond to the aryl radical. While the indolyl side chain of tryptophan has the advantage of being more reactive toward aryl radicals than the phenolic side chain of tyrosine, its major drawback is the possible competition of azo coupling of aryldiazonium ions with the indole subunit.

Starting with relatively conventional methods involving stoichiometric amounts of an oxidant or reductant, this overview shows that catalytic and even additive‐free arylations may also be highly efficient. In particular, among the most recently published methods, photochemistry plays a major role.

Most interesting, however, is, in our opinion, the remarkable selectivity that could already be achieved in radical arylations of diverse, highly complex peptidic substrates. In particular, these transformations underline the value of aryl radicals in peptide functionalization and can serve as a promising starting point for the development of further radical arylation methods as well as for their extended application to complex peptidic substrates, thereby enabling straightforward access to novel peptides for diverse biological investigations as well as for drug discovery.

## Funding

Deutsche Forschungsgemeinschaft (DFG‐HE5413/10‐1).

## Conflicts of Interest

The authors declare no conflicts of interest.

## Data Availability

Data sharing not applicable to this article as no datasets were generated or analyzed during the current study.
